# Recompression in new levels after percutaneous vertebroplasty and kyphoplasty compared with conservative treatment

**DOI:** 10.1007/s00402-013-1886-3

**Published:** 2013-11-28

**Authors:** Xiaodong Yi, Hailin Lu, Fei Tian, Yu Wang, Chunde Li, Hong Liu, Xianyi Liu, Hong Li

**Affiliations:** Department of Orthopaedic Surgery, Peking University First Hospital, Xicheng district, Bejing, 100034 China

**Keywords:** Percutaneous vertebroplasty, Kyphoplasty, Adjacent fracture, Conservative treatment

## Abstract

**Study design:**

A prospective clinical study assessing new vertebral compression fracture after previous treatment.

**Objective:**

The purpose of this study was to investigate the incidence and associated risk factors of new symptomatic osteoporotic vertebral compression fractures (OVCFs) in patients treated by percutaneous vertebroplasty (PVP) and kyphoplasty (PKP) versus conservative treatment, and to elucidate our findings.

**Summary of background data:**

There are a lot of reports concerning the feasibility and efficacy of this minimally invasive procedure compared with conservative treatment, especially in pain soothing. However, it is still unclear whether the risk of subsequent fracture has increased among operative treatment patients in the long term.

**Methods:**

From November 2005 to July 2009, 290 consecutive patients with 363 OVCFs were randomly selected for PVP/PKP or conservative treatment and evaluated with a mean follow-up of 49.4 months (36–80 months). Some parameters were characterized and statistically compared in this study. Telephone questionnaires, clinical reexamine, and plain radiographs were performed in the follow-up.

**Results:**

Thirty-one of 290 (10.7 %) patients had experienced 42 newly developed symptomatic secondary OVCFs. Among 169 operation (53.3 % vertebroplasty, 46.7 % kyphoplasty) and 121 comparison patients, there is no significant statistical difference of new OVCFs incidence between the two groups calculated by patient proportion. However, in separate, the rate of secondary adjacent fractures calculated by vertebral refracture number is significantly higher than non-adjacent levels in PVP/PKP group but no significant statistical difference was observed in conservative group. The time interval of recompression after operative procedure was much shorter than that for comparison group (9.7 ± 17.8 versus 22.4 ± 7.99 months, *p* = 0.017). In addition, older age, gender, fracture times, location of original fracture segment, the amount of cement, cement leakage, operation modality (PVP or PKP),and initial number of OVCFs were documented, but these were not the influencing factors in this study (*p* > 0.05).

**Conclusions:**

Patients who had experienced PVP/PKP were not associated with an increased risk of recompression in new levels. However, recompression in new levels of PVP/PKP group occurred much sooner than that of conservative group in the follow-up period. The incidence of new vertebral fractures observed at adjacent levels was substantially higher but no sooner than at distant levels in PVP/PKP group. No major risk factors involving new OVCFs have been found in this study and  augmentation for sandwich situation is not necessary.

## Introduction

Since 1987, percutaneous vertebroplasty (PVP) and kyphoplasty (PKP) with polymethylmethacrylate (PMMA) augmentation have been highly advocated as treatment techniques for osteoporotic vertebrate compression fractures (OVCFs) [1]. Although the overall satisfaction rate ranges between 70 and 90 %, and complication rate associated is reported to be very low, some authors have indicated that PVP/PKP increases the risk for subsequent vertebral fractures [2–5].

Multiple covariate analysis, such as patient characteristics and procedural techniques, has been used to identify risk factors [6–7]. But it is still unclear as to what the exact relevant element is and whether such fractures are procedure-related or part of the natural course of osteoporosis. There also have been no reports to date of a large series of new vertebral fractures that developed within adjacent or nonadjacent levels comparing PVP/PKP and conservative treatment. In our original study, we prospectively investigate the incidence of new symptomatic OVCFs in patients treated by PVP/PKP or conservative therapy and discuss the possible causative mechanism of refracture.

## Materials and methods

Our study was performed at the Peking University First Hospital Orthopedics Department and approved by Institutional Review Board. Herein we report our first hand data statistics regarding China mainland.

### Study design

From November 2005 to July 2009, a total of 290 patients with 363 symptomatic OVCFs received treatment at our institution. Patients were randomized to one of three different treatments: percutaneous vertebroplasty (PVP) (*n* = 90), and kyphoplasty (PKP) (*n* = 79) and conservative treatment (CT) (*n* = 121). All patients underwent bone density examination before treatment and the extent of osteoporosis was not significantly different among them. The study was designed to investigate the incidence of recompression in adjacent or distant levels after conservative treatment compared with PVP/PKP and also to detect risk factors. The following were the inclusion criteria: symptomatic OVCFs at first time with serious low back pain and a high signal in T2 MRI image, diagnosed by severe osteoporosis.

A surgeon at the outpatient ward blindly chose one of the three different treatment modalities to ensure similar pre-treatment age, symptoms, grade and level of spinal diseases among the patients. One senior surgeon performed all the operations and another spine surgeon not involved with the surgeries analyzed the results.

No patients were lost to follow-up during the study. There were 181 women and 109 men and the average patient age at the time of treatment was 61.3 years (age range, 48–75 years). The average duration of follow-up was 49.4 months (range 36–80 months).

#### Operation group

We performed operation in 217 vertebrae for 169 patients, 53.3 % (90) with vertebroplasty and 46.7 % (79) with kyphoplasty. PVP/PKP was not performed in case of a severe spinal stenosis with infiltration of the posterior wall or with any neurological deficit, coagulopathy, infection, radicular syndrome, and some other contraindications. Patients who were treated for metastatic disease, corticosteroid-induced subsequent osteoporosis, vertebral hemangioma or treated in combination with surgery were also excluded. Since there is no selection bias in our institution regarding treatment with PVP or PKP, we combine them into one group as compared with conservative group.

#### Conservative group

The other 121 patients diagnosed by similar baseline characteristics with PVP/PKP group received conservative treatment. The exclusion criteria were also the same with PVP/PKP group.

### Surgical technique and clinical intervention

**Fig. 1:**
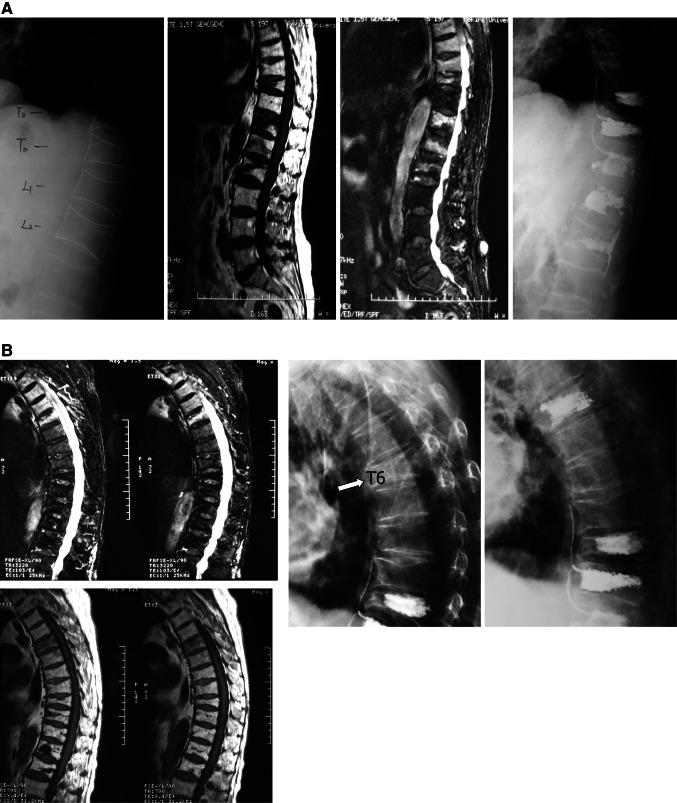
**a** Sagittal T1, T2-weighted MR and plain images show T10, T12–L2 OVCF of a 73-year-old man treated by PKP in February, 2008. T11 was treated prophylactically. **b** Ten months later, a new compression fracture at T6 was subjected to repeated PKP

Every PVP/PKP-treated patient received local anesthesia and intravenous conscious sedation during the procedure. An 11-G bone marrow biopsy needle (REF T05E kyphx Osteo-Introducer System, kyphon Inc. Medtronic, Minnesota) was used to puncture the collapsed vertebral body through either side of the pedicles, and the needle advanced to the anterior third of the vertebral body under bi-plane fluoroscopic guidance (Arcardis Varic mobile X-ray system, Siemens, Germany). After transpedicular positioning, the needles were exchanged over a guidewire for a working cannula (For PVP, we choose Kynefyc systems, Shanghai, China). For PKP, inflatable bone tamps (REF K02A, kyphx Xpander Inflatable Bone Tamp Kyphon, Medtronic, Minnesota) were then placed into the vertebral bodies unilaterally. A dedicated delivery system (REF F04B, kyphx Bone Filler Device, kyphon Inc. Medtronic, America) was used to inject the bone cement. In the case of “sandwich bodies” (f.i. L2 and L4 to be treated), some intermediate vertebral bodies were prophylactically treated (Fig. 1). Usually pain was relieved almost immediately after the procedure: patients could mobilize themselves several hours later and no brace was needed. Overnight hospital stay was required and patients were discharged home the next day. Patients in the conservative group were offered pain medication, bed rest, a solf bi-valved body brace, and physiotherapy; the mean length hospital stay was 13.7 days.

A series of anti-osteoporosis therapies were applied to treat osteoporosis and prevent refracture in both groups. The available pharmacologic therapy included bisphosphonates, calcium carbonate particles, salmon calcitonin injection, and calcitriol soft capsules, which were advised to be taken at least for 2 years. Tobacco cessation, restrained alcohol consumption, weight-bearing exercise, and fall prevention strategy were also required to be complied with in the follow-up period.

### Radiological and clinical assessment

All the patients had undergone radiography and MRI of the lumbar spine before treatment to diagnose the symptomatic OVCFs and to evaluate the status of an adjacent segment. For validation, measurements were performed twice by three independent observers, two experienced spine surgeons and one radiologist, to ensure randomization of treatment method. Radiographs included standing anteroposterior and lateral views. The same radiographs and MRI were repeated at 6 months and at yearly intervals until the last follow-up session. If high T2 MRI signal was observed in new segments and A VAS score >7 was identified, new symptomatic OVCFs were defined excluding other diseases, so as to refracture of the same index segment. In this study, we only recorded and analyzed the symptomatic OVCFs.

### Statistical analysis

We performed statistical analysis with SPSS for Windows Version 16.0 (SPSS, Chicago, IL, USA). Chi square test was used to compare the incidence and patient proportions. The paired *t* test was used for evaluation of differences between adjacent and nonadjacent segments. Data are expressed as mean ± SD. A *p* value of <0.05 was considered statistically significant.

## Results

No major adverse events were observed during procedure or follow-up period. 87.6 % patients of PVP/PKP group returned to their normal lives 1 week after operation and 59.2 % patients of conservative group exercise to their regular level 2 months later. In total, 31 of 290 (10.7 %) patients returned with 42 new OVCFs (Table 1). 14 individuals (5 males, 9 females) were initially treated by surgical technique (PVP 9, PKP 5) and 17 ones (7 males, 10 females) were from conservative group. 25 patients were subjected to repeated PVP/PKP and the other six patients were successfully managed by conservative care. Of these newly documented fractures, 14 (34.2 %) involved adjacent segments, whereas 25 (59.5 %) occurred at distant levels, and another three (7.3 %) were the same.

**Table 1:** Baseline clinical features of 31 patients who experienced new OVCF

Age	Gender	Level(s) of initial OVCFs	Time interval of recompression	Level(s) of new OVCFs	Initial operating modus	PMMA amount per vertebra
65	M	L1	23 months	T11T12	PVP	4
76	M	L1 L2	11 months	T11T12	PVP	4.5 3
89	M	L2 L3	9 months	L1L4	PVP	3 3
79	M	L2	9 months	L1	PVP	3.5
53	F	T7	23 months	L2	PVP	4
65	F	T11	3 months	L1L2	PVP	3.5
76	F	T12	16 months	L1	PVP	6
76	F	L2	1 months	L1	PVP	5
79	F	T5 T6 T7	2 months	L2	PVP	1 1 1
75	F	L2	22 months	T12	PKP	5
73	M	T10–L2	10 months	T6	PKP	2.5 3 2 5 1.5
79	F	T7	2 months	T8	PKP	1
78	F	L1	20 days	T12	PKP	5
60	F	L1	4.5 months	L4	PKP	3
72	F	L1	34 months	T12	CT	n.a.
79	F	L1	26 months	T12	CT	n.a.
76	M	T6	26 months	T5	CT	n.a.
66	M	T9 T12 L1 L3	12 months	L1	CT	n.a.
49	M	T12	72 months	L1	CT	n.a.
63	M	L4 L5	8 months	L2T12	CT	n.a.
77	M	T10	20 days	T12	CT	n.a.
63	M	T8	7.5 months	L1	CT	n.a.
61	M	T6	32 months	L2	CT	n.a.
79	F	T8	36 months	T12	CT	n.a.
75	F	T11	24 months	L2	CT	n.a.
74	F	T9	36 months	T12	CT	n.a.
65	F	T8	10 months	L1L2L5	CT	n.a.
72	F	T9	5 months	T9-12	CT	n.a.
54	F	L4 L5	12 months	T10	CT	n.a.
79	F	T12 L2–4	48 months	T6T9	CT	n.a.
78	F	L1 L3	5 months	L1	CT	n.a.

*CT* conservative therapy, *n*.*a*. not applicable

### PVP/PKP group

In terms of frequency of recompression, we selected a method similar to that of Dr Johannes et al. [8]. Fourteen (8.28 %) of these 169 patients developed 18 new OVCFs, three (21.4 %) of the 14 patients had a third compression fracture and a third PVP. Excluding the cervical spine as well as TH1–TH2 segments due to low risk of OVCFs, the other 15 levels (TH3–L5) were considered to occur new fracture(s) potentially. In the PVP/PKP group, 217 bodies were intervened initially and 18 (0.71 %) of the whole 2,535 levels (169 patients × 15 vertebrae) developed new OVCFs, nine of them belonged to adjacent segments, the other nine belonged to distant segments, none of the pretreated segments recollapsed. Of these 2,318 (2,535–217) vertebral bodies, 360 (15.5 %) were adjacent to pretreated segments, while 1,958 levels came from the distant. Therefore, the percentages of new adjacent/distant OVCFs were 2.5 % (9/360) and 0.46 % (9/1,958) for the second compression fractures (*χ* ^2^ = 16.4, *p* = 0.00). For the third new OVCFs, the data were 5.12 and 1.53 %, respectively (*p* = 0.239). Adjacent fractures were more prevalent than distant ones for the second fractures; however, the differences had not reached statistical significance for the third-time fractures. Of the 18 new fracture segments, ten new fracture segments were from levels above the pretreated segments, the other eight were from levels below the pretreated segments, and no refracture of pretreated segments was detected. The distance from the initial fracture segment in distant group was 2, 2, 2, 2, 3, 3, 4, 7, 7 level(s), respectively. Calculated by patient number, six patients had new adjacent fractures and the other six was from nonadjacent cluster, with two patients experienced both adjacent and non-adjacent fractures.

The mean age of the 155 patients was 70.9 years, a little younger than the refracture unit (73.1 ± 8.93, *t* = 1.974, *p* = 0.43). Males comprised 31.6 % (51 cases), and in the refracture group it was 35.7 % (5 cases, *p* = 1.00).

The distribution of original and new OVCFs is shown in Table 2. New fracture(s) occurred mostly in the thoracic and thoracolumbar segment compared with the previous fracture(s), but difference was not statistically significant (*t* = 2.11, *p* = 1.00).

**Table 2:** Distribution of initial and new OVCFs

Level	PVP/PKP initial	PVP/PKP new	Conservative initial	Conservative new
T3–T7	13 (5.99 %)	1 (5.56 %)	10 (6.85 %)	2 (8.33 %)
T8–T10	15 (6.92 %)	1 (5.56 %)	20 (13.7 %)	5 (20.8 %)
T11	14 (6.45 %)	2 (11.11 %)	11 (7.53 %)	1 (4.16 %)
T12	39 (17.97 %)	4 (22.22 %)	24 (16.5 %)	6 (25.0 %)
L1	60 (27.65 %)	5 (27.77 %)	35 (23.9 %)	5 (20.8 %)
L2	37 (17.05 %)	3 (16.67 %)	23 (15.75 %)	4 (16.7 %)
L3	17 (7.84 %)	0 (0 %)	6 (4.11 %)	0 (0 %)
L4	16 (7.37 %)	2 (11.11 %)	9 (6.17 %)	0 (0 %)
L5	6 (2.76 %)	0 (0 %)	8 (5.49 %)	1 (4.16 %)
Sum	217	18	146	24

**Fig. 2:**
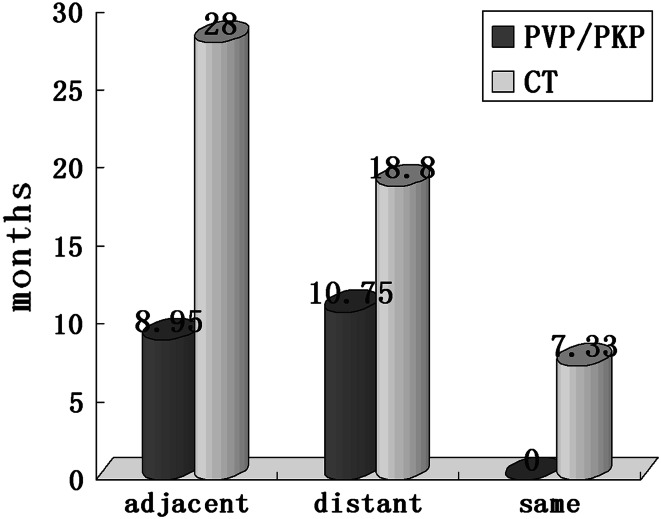
New OVCFs occurred sooner after PVP/PKP than after conservative treatment. Same level refractures received conservative treatment occurred much sooner than any other units

The time interval was 8.95 ± 7.34 months for adjacent group and 10.75 ± 8.68 months for nonadjacent group. It seems that adjacent new OVCFs occurred earlier than nonadjacent levels; however, we did not find a statistically significant difference (*t* = 1.548, *p* > 0.05). Of the three patients that experienced third OVCFs,the symptom-free interval after a second compression fracture was 6, 2 months for adjacent segments and 15 months for a patient in the same treated segment. Owing to limited number of third fracture cases, we could not compare the data effectively (Fig. 2).

The mean number of preexisting fractures in single time fracture group was 1.36 (1.36 ± 0.75, 95 %CI, 1.24–1.47) and in repeated fracture group, 1.57 (1.57 ± 1.11, 95 %CI, 0.92–2.21). As can be seen from the above, baseline number of vertebra fracture is not a predictor for new fractures (*t* = 1.972, *p* > 0.05). The amount of injected cement was 3.95 ± 1.43 (range 1.5–9 mL) in single time fracture group and 3.26 ± 1.54 (range 1–6 mL) in the repeated fracture group. There was no positive association between the quantity and recollapse of new segment (*t* = 1.4651, *p* > 0.05).

PMMA leakage was observed in four cases (1.77 %) after the first incident: two into the disk, one into the lateral blood plexus and one with anterior extravasation going up the above level (Fig. 1b T11). None of the leaks exuded into neural canal or caused neurologic symptoms. None of the patients developed refracture.

### Conservative group

During the same follow-up period, 17 (14.0 %) of the 121 patients went through 24 (24/1, 815,1.32 %) new OVCFs, two (11.7 %) developed a third compression fracture and a third PKP. Five segments belonged to adjacent section, 16 from nonadjacent ones, and the remaining 3 from the same previous fracture segments. Using the same calculating method with PVP/PKP group, the proportion of adjacent new fractures (1.91 %, 5/261) is also higher than nonadjacent fractures (1.13 %, 16/1,408), but did not reach statistical significance (*χ* ^2^ = 0.519, *p* = 0.471). Eight segments occurred above pretreated levels; thirteen from below sites, while 3 were from the same segments.

As the number of third fracture was limited, it was difficult to monitor the relative risk of new fractures sufficiently. With the accretion of distance from the original fracture level, recompression incidence decreased gradually. The mean time interval was 22.4 ± 7.99 months (range 0.67–72 months). It seems that fracture occurrence in adjacent segments was more delayed than nonadjacent segments (28 ± 3.46 versus 18.8 ± 14.3 months);however, the data had not reached statistical significance.(*t* = 1.361, *p* > 0.05). The original and new OVCFs group also did not differ significantly in age, gender distribution, location of original fracture segments, and number of preexisting fractures.

### Comparison between PVP/PKP group and conservative group

We exclusively compared the data of second refractures due to only a minority of third recompressions. From our data, incidence of recompression in the conservative group is higher than that in the PVP/PKP group, with 14.05 % compared with 8.28 % concerning patient proportion and 1.32 % compared with 0.71 % regarding refracture number. The relative risk is 1.69, but no statistical significance has been reached (*χ* ^2^ = 2.455, *p* = 0.117). Both groups indicate a higher incidence of adjacent recompression; however, the relative risk in PVP/PKP group (2.5 %, 9/360) is not significantly higher than that in conservative group (1.91 %, 5/261, *p* = 0.628).

Most of the second fractures in the PVP/PKP group occurred within the first year (71.42 %) compared with less than half of the recompression in the conservative group (47.05 %),and the mean interval was also much shorter in PVP/PKP group (9.72 ± 17.8 months) than that in the conservative group (22.48 ± 7.99 months, *p* = 0.017). Adjacent and nonadjacent new OVCFs of PVP/PKP group also occurred remarkably sooner than in the counterparts of conservative group. No major differences were observed in other parameters as presented in Table 3.

**Table 3:** Baseline characteristics of patients in PVP/PKP group and conservative group

Parameter	PVP/PKP once	PVP/PKP twice	Conservative therapy once	Conservative therapy twice	P1	P2	P3
No (patients)	155	14	104	17	n.a.	n.a.	n.a.
Age, mean ± SD (range)	70.9 ± 10.04 (43–97)	73.1 ± 8.93 (53–89)	63.9 ± 15.51 (11–92)	69.5 ± 8.92 (49–79)	0.43	0.159	n.a.
Sex, female (%)	104 (68.4 %)	9 (64.2 %)	58 (55.7 %)	10 (58.8 %)	1.00	0.814	n.a.
Original fracture site (vertebrae)							
Thoracic spine	71 (36.6 %)	10 (43.4 %)	52 (43.3 %)	13 (50 %)	0.519	0.535	n.a.
Lumbar spine	123 (63.4 %)	13 (56.6 %)	68 (56.7 %)	13 (50 %)			
Amount of bone cement	3.95 ± 1.43 (range 1.5–9 mL)	3.26 ± 1.54 (range 1–6 mL)	n.a.	n.a.	0.22	n.a.	n.a.
PMMA leakage	3 cases	n.a.	n.a.	n.a.	n.a.	n.a.	n.a.
Number of previous fracture							
Mean	1.36 ± 0.75	1.57 ± 1.11	1.20 ± 0.46	1.53 ± 0.97	0.23	0.21	n.a.
One	130	9	86	12			
Two	15	3	15	3			
Three	6	1	3	0			
Four	4	0	0	2			
Five	0	1	0	0			
Distribution of new OVCFs							
Total	n.a.	18 (0.71 %)	n.a.	24 (1.32 %)	n.a.	n.a.	0.042
Adjacent	n.a.	9 (2.5 %)	n.a.	5 (1.91 %)	n.a.	n.a.	0.117
Between	n.a.	n.a.	n.a.	n.a.	n.a.	n.a.	n.a.
Distant	n.a.	9 (0.46 %)	n.a.	16 (1.13 %)	n.a.	n.a.	0.025
Same	n.a.	n.a.	n.a.	3 (2.05 %)	n.a.	n.a.	n.a.
Below	n.a.	8	n.a.	13	n.a.	n.a.	n.a.
Above	n.a.	10	n.a.	8	n.a.	n.a.	n.a.
Incidence (patients)							
Total	n.a.	14 (8.28 %)	n.a.	17 (14.05 %)	n.a.	n.a.	0.117
Adjacent	n.a.	8 (4.73 %)	n.a.	5 (4.13 %)	n.a.	n.a.	0.903
Distant	n.a.	8 (4.73 %)	n.a.	11 (9.09 %)	n.a.	n.a.	0.130
Same	n.a.	n.a.	n.a.	3 (2.48 %)	n.a.	n.a.	n.a.
Distance from the original fracture site							
One level	n.a.	9	n.a.	5	n.a.	n.a.	n.a.
Two levels	n.a.	4	n.a.	4	n.a.	n.a.	n.a.
Three levels	n.a.	2	n.a.	4	n.a.	n.a.	n.a.
Four levels	n.a.	1	n.a.	2	n.a.	n.a.	n.a.
Five levels	n.a.	0	n.a.	2	n.a.	n.a.	n.a.
Six levels	n.a.	0	n.a.	2	n.a.	n.a.	n.a.
Seven levels	n.a.	2	n.a.	1	n.a.	n.a.	n.a.
Eight levels	n.a.	n.a.	n.a.	1	n.a.	n.a.	n.a.
Time interval (months)	n.a.	9.72 ± 17.8	n.a.	22.4 ± 7.99	n.a.	n.a.	0.017
Adjacent	n.a.	8.95 ± 7.34	n.a.	28 ± 3.46	n.a.	n.a.	0.0002
Distant	n.a.	10.75 ± 8.68	n.a.	18.8 ± 14.3	n.a.	n.a.	0.255
Same	n.a.	n.a.	n.a.	7.33 ± 3.29	n.a.	n.a.	n.a.
Above	n.a.	10.71 ± 7.74	n.a.	25.6 ± 13.3	n.a.	n.a.	0.031
Below	n.a.	8.5 ± 7.53	n.a.	20.0 ± 11.7	n.a.	n.a.	0.058
Access							
Unilateral	213	23	n.a.	n.a.	n.a.	n.a.	n.a.
Bilateral	4	0	n.a.	n.a.	n.a.	n.a.	n.a.
Procedure							
PKP	79	5 (6.33 %)	n.a.	n.a.	n.a.	n.a.	n.a.
PVP	90	9 (10 %)	n.a.	n.a.	n.a.	n.a.	n.a.

P1 for the difference between single time fracture group and multifracture group after PVP/PKP

P2 for the difference between single time fracture group and multifracture group after conservative treatment

P3 for the difference in refracture group between PVP/PKP and conservative treatment

n.a. not applicable

After the initial treatment, a total of 27 sandwich situations were created in both groups, no sandwich fracture was observed in our follow-up period. Time interval distinction between above and below levels after PMMA injection also did not reach statistical significance (10.71 ± 7.74 versus 8.5 ± 7.53 months, *p* = 0.62), so did conservative therapy (25.6 ± 13.3 versus 20.0 ± 11.7 months, *p* = 0.45).

## Discussion

A total of 1.5 million new fractures, nearly half of which are vertebral (700,000), are reported in the US each year, outnumbering fractures of the hip and ankle combined. Patients with osteoporotic vertebral compression fractures often suffer from acute episodes of pain, increased morbidity, prolonged hospitalization as well as long-term disability, as the natural healing history of OVCFs is often accompanied by 1–3 months bedrest. Also, the risk of pain and disability increases progressively with the number and severity of vertebral deformities. They are not typically associated with neurological deficit, since the fractures do not extend into the spinal canal nor compress the neural elements. Generally, acute pain resolves in 4 weeks to 8 months. In the years before PVP/PKP invention, conservative management of OVCFs consists of bed rest, analgesic medications, bracing, and physical therapy. However, some patients do not respond to these therapies in either terms of pain relief or progression of deformity. Long-term staying in bed can also bring out a series of complications, such as pneumonia, anorexia, indigestion, malnutrition, even stroke. An ideal therapy should both address fracture-related pain and avoid the associated complications in a minimally invasive fashion. Since 1990s, PVP/PKP had become standard procedures for OVCFs regarding effective pain reduction and relatively low incidence of complications. But recently, many clinical investigations have reported an increased fracture risk of adjacent levels. Hulme et al. [9] reviewed 69 clinical series evaluating both kyphoplasty and vertebroplasty: 766 patients after PKP had 115 new fractures and 66 % were located at adjacent levels. Mazzantini et al. [10] found a 27.8 % cumulative incidence of new vertebral fractures in a cohort of 115 patients treated with PVP after a follow-up of mean 39 months; 68 % occurred next to pretreated segments. Tseng et al. [11] investigated 852 patients (1,131 vertebrae),141 patients (16.6 %) after PVP experienced new OVCFs, 58.8–63.8 % of which were adjacent fractures. Similarly, Trout et al. [12] also reported a significant increase (41.4 %) in the incidence of fractures in the vicinity of cemented vertebrae on evaluation of 432 patients.

Unfortunately, much of the current literature reports their studies comparing original fractures and new fractures mainly in operative group, there are only several limited data with respect to recompression incidence of PVP/PKP versus conservative treatment. Rikke et al. [13] reported that the relative risk for all new fractures in the PVP group compared with the conservative group was 2.9 after 3 months and 1.3 after 12 months. However, Klazen et al. [14] observed 202 patients; there was no difference between PVP and conservative therapy.

In the current study, we found a similar incidence of recompression comparing PVP/PKP (8.28 %) with conservative therapy (14.0 %) on accordance of patient number, which is coincident with Klazen’s conclusion. From the standpoint of vertebrae, adjacent recompression occurred more frequently than distant levels and decreased gradually with the accretion of intervals in the PKP/PVP group. By contrast, no higher incidence of recompression in adjacent levels was observed in the conservative group. Prospective and retrospective studies reported an incidence of new fractures of 12.5–36.8 % after PVP/PKP [10, 15, 16]; our data seem to be favorable and could be the consequence of long-term compliance. In addition, we only registered symptomatic fractures, whereas in most of higher rate reports recompression was defined by imaging follow-up. Moreover, in our experience, we intended to only fill the cavity left by the balloon or the fracture fissure rather than augment the entire vertebral body. Most patients followed our guidelines for anti-osteoporosis therapy, which may also interpret the difference in refracture ratio. Tanigawa et al. [16] reported that 56 new OVCFs occurred in 28 (36.8 %) of 76 patients after PVP, and 38 (67.8 %) of these fractures occurred in adjacent levels, more than half occurred within 3 months.

The exact mechanism for recompression is still unclear; several authors had published their clinical and biomechanical investigations. Most of them indicate that load transfer mechanism after augmentation is remarkably changed with increased stresses and strains in the levels of vicinity. Michael et al. [17] found the depression of the fractured endplate altered the pressure profile of the damaged disk resulting in increased compressive loading of the anterior wall of adjacent vertebra that predisposed it to wedge fracture. Rohlmann et al. [18] also suggest that a wedge-shaped fracture increases the flexion bending moment due to the upper body weight and thus a higher muscle force in the erector spinae is required to balance the spine, which results in a higher spinal load and a higher intradiscal pressure. The erector spinae is a long muscle and thus its force affects intradiscal pressure not only at adjacent levels but also the whole region. Our findings agreed with this contention for the location of refracture body all residing in the vicinity of eight levels. Viewed from this point, kyphoplasty may be an ideal alternative method in preventing further collapse compared with vertebroplasty for its rectification of kyphotic deformity. In this study, we observed that refracture incidence in PKP group is lower than that in PVP group and the conservative group (6.33 versus 10 %, 14.05 %, *p* = 0.426, 0.088), but not significantly. Most clinical and basic findings [19–21] asserted that the rate of developing new fractures was not strongly influenced by the volume of cement injected, as in the present study the average amount of initial bone cement is similar between single time fracture unit and refracture unit in the PVP/PKP group (3.95 ± 1.43 versus 3.26 ± 1.54 mL, *p* = 0.22).

In our current report, the rate of refracture concerning adjacent bodies is truly significantly higher than distant ones in PVP/PKP group (2.5 versus 0.46 %, *p* = 0.000), but the difference is not conspicuous in the conservative group (1.91 versus 1.13 %, *p* = 0.471). Nevertheless, the unitary potentiality in the PVP/PKP group is inversely lower than in the conservative group (0.71 % versus 1.32 %, *p* = 0.042) from the standpoint of vertebrae. From these data we can extrapolate that both previous fracture and cement injection procedure increase the risk of new adjacent fractures, but cement injection procedure increases the risk more significantly. However, the odds is relatively low and has no significant differences compared with conservative treatment (2.5 versus 1.91 %, *p* = 0.636). Therefore, patients who experienced PVP/PKP may have a relatively low risk of developing new fractures, not to mention its immediate pain relief, early mobilization, and other short-term benefits.

The timing of postoperative OVCFs in our study also seems to be more favorable than that in prior studies, which have shown that between 43 and 67 % of subsequent incident fractures occur within 3 months. In the PVP/PKP group, 71.42 % of the second fractures take place within the first year, while only 47.05 % of the second fractures in the conservative group do in the same period. Mean interval was much shorter in PVP/PKP group (9.72 ± 17.8 months) than in conservative group (22.48 ± 7.99 months, *p* = 0.017). Both Tseng et al. [11] and Trout et al. [12] asserted that adjacent fractures emerged sooner than distant levels following cement injection. The period from a fresh compression fracture to the first adjacent compression fracture was 78.2 ± 97.1 days and to the first distant compression fracture was 231.9 ± 224.6 days according to Tseng et al. While in our clinical follow-up a similar result was observed in the PVP/PKP group, the data are 8.95 ± 7.34 and 10.75 ± 8.68 months, respectively (*p* < 0.05). For conservative treatment, the new adjacent segments occurred even more delayed than nonadjacent segments although no statistical discrepancy has been reached (28 ± 3.46 versus 18.8 ± 14.3 months, *p* > 0.05). So we can hypothesize that cement injection may precipitate the refracture, particularly to adjacent segments.

No same level refracture was observed following PVP/PKP, while in conservative group, the refracture rate was relatively high (2.05 %) and occurred sooner than that in any other unit (7.33 ± 3.29 months). Thus usage of operation procedure may be advantageous in holding back further refracture of the same segment. However, even cemented vertebrae refracture occasionally, Lin et al. [6] reported an incidence of 63 % in their retrospective clinical analysis, and concluded that significant anterior vertebral height restoration increases the risk of subsequent fracture after vertebroplasty. This conclusion challenges some theories we have discussed above; one problem is how to guarantee enough kyphotic angle rectification on the one hand while avoiding greater anterior vertebral height restoration on the other.

Johannes et al. [8] demonstrated a remarkable propensity of refractures within three levels above or below preexisting fractures. From our data, we got a similar outcome; 83.3 % of new OVCFs in PVP/PKP group and 56.2 % in conservative group support this interpretation. The locations in thoracolumbar segments where original fractures distributed in are prone to refracture both after PMMA injection and conservative therapy. New fractures tend to occur more frequently in thoracic vertebrae in both groups but no conspicuous discrepancy. The issue of multitime fractures (>2) after PVP has been raised and analyzed to some extent by Tseng et al. [11]. In our study these data were so limited and, therefore, could not be compared.

Another risk factor of new fractures of adjacent vertebral bodies is cement leakage into the disk. Pitton et al. [22] studied 191 patients in whom 385 vertebroplasties were performed. The overall rate of cement leaks was 55.6 %, including all leaks detectable by CT. Ten of 30 adjacent fractures occurred in the presence of preexisting intradiscal cement leaks and six belonged to 11 sandwich fractures of 29 sandwich situations after mean 1.5 months. However, in three cases (1.77 %) cement leakage occurred during operation in our institution and none developed refracture. In the meantime, no sandwich fracture was observed in a total of 27 sandwich situations in both groups. Pitton et al. identified that the sandwich situation changed the contextual environment because of the stiffening and the loss of vertical elasticity of the two proximate vertebrae but was not associated with an increased secondary fracture rate. Our data support their viewpoint, yet no evidence is available until now. Further researches are needed to focus on this specific constellation. In addition, older age, gender, fracture times, operation modality (PVP or PKP), and initial number of OVCFs were also documented, but they were not the influencing factors in developing new symptomatic OVCFs in this study.

We selected a calculating method similar to that of Johannes et al. [8], in which adjacent and nonadjacent segments were divided, because the total number of adjacent vertebrae generated by first time fracture is relatively small compared with that of distant levels. As a result, only simply computing the incidence of adjacent refractures by dividing the sum number of new OVCFs cannot reflect the condition practically. However, if count the refracture incidence by simply calculating the patient number, the above two therapeutics could bring out similar results.

Some issues remain unanswered even in the current study. We discussed the effects of PVP/PKP and conservative therapy only clinically, not biomechanically, and long-term effects are still unknown. Furthermore, whether resort to cement injection or let it alone in terms of sandwich situation is not known. Therefore, further research using larger patient populations and in vitro experiment may help to resolve the unclear results concerning this study. Moreover, the results of this study need to be confirmed by additional high-quality RCT clinical studies.

## Conclusions

From most data reported in the literatures, the risk of experiencing new OVCFs increased after PVP/PKP. However, in this prospective study, compared with conservative therapy, patients who had experienced PVP/PKP were not associated with an increased risk of recompression in new levels with a mean 49.4-month follow-up period. But one point that new OVCFs occurred sooner after cement injection than that after conservative treatment must be taken into consideration. The presence of sandwich situation is not associated with an increased secondary fracture rate and no interrelated factor has been confirmed to be influential ingredient for subsequent fractures. Existing fractures are strong independent predictors of the risk of future vertebral fractures so we tend to believe that it is more of a natural process other than induced by PVP/PKP.

## References

[1] Galibert P, Deramond H, Rosat P (1987). Preliminary note on the treatment of vertebral angioma by percutaneous acrylic vertebroplasty. Neurochirurgie.

[2] Lindsay R, Silverman SL, Cooper C (2001). Risk of new vertebral fracture in the year following a fracture. JAMA.

[3] Ross PD, Genant HK, Davis JW (1993). Predicting vertebral fracture incidence from prevalent fractures and bone density among non-black, osteoporotic women. Osteoporos Int.

[4] Pflugmacher R, Schroeder RJ, Klostermann CK (2006). Incidence of adjacent vertebral fractures in patients treated with balloon kyphoplasty: two years’ prospective follow-up. Acta Radiol.

[5] Uppin AA, Hirsch JA, Centenera LV (2003). Occurrence of new vertebral body fracture after percutaneous vertebroplasty in patients with osteoporosis. Radiology.

[6] Lin WC, Lee YC, Lee CH (2008). Refractures in cemented vertebrae after percutaneous vertebroplasty: a retrospective analysis. Eur Spine J.

[7] Komemushi A, Tanigawa N, Kariya S (2006). Percutaneous vertebroplasty for osteoporotic compression fracture: multivariate study of predictors of new vertebral body fracture. Cardiovasc Intervent Radiol.

[8] Johannes H, Heiko F, Kerstin W (2006). Incidence of symptomatic vertebral fractures in patients after percutaneous vertebroplasty. Cardiovasc Intervent Radiol.

[9] Hulme PA, Krebs J, Ferguson SJ (2006). Vertebroplasty and kyphoplasty: a systematic review of 69 clinical studies. Spine.

[10] Mazzantini M, Carpeggiani P, d’Ascanio A (2011). Long-term prospective study of osteoporotic patients treated with percutaneous vertebroplasty after fragility fractures. Osteoporos Int.

[11] Tseng YY, Yang TC, Tu PH (2009). Repeated and multiple new vertebral compression fractures after percutaneous transpedicular vertebroplasty. Spine.

[12] Trout AT, Kallmes DF, Kaufmann TJ (2006). New fractures after vertebroplasty: adjacent fractures occur significantly sooner. AJNR Am J Neuroradiol.

[13] Rikke R, Karina LH, Mikkel OA (2010). Twelve-months follow-up in forty-nine patients with acute/semiacute osteoporotic vertebral fractures treated conservatively or with percutaneous vertebroplasty. Spine.

[14] Klazen CA, Venmans A, de Vries J (2010). Percutaneous vertebroplasty is not a risk factor for new osteoporotic compression fractures: results from VERTOS II. AJNR Am J Neuroradiol.

[15] Kim YY, Rhyu KW (2010). Recompression of vertebral body after balloon kyphoplasty for osteoporotic vertebral compression fracture. Eur Spine J.

[16] Tanigawa N, Komemushi A, Kariya S (2006). Radiological follow-up of new compression fractures following percutaneous vertebroplasty. Cardiovasc Intervent Radiol.

[17] Michael NT, Susan MR, Frank MP (2008). Altered disc pressure profile after an osteoporotic vertebral fracture is a risk factor for adjacent vertebral body fracture. Eur Spine J.

[18] Rohlmann A, Zander T, Bergmann G (2006). Spinal loads after osteoporotic vertebral fractures treated by vertebroplasty or kyphoplasty. Eur Spine J.

[19] Berlemann U, Ferguson SJ, Nolte LP (2002). Adjacent vertebral failure after vertebroplasty: a biomechanical investigation. J Bone Joint Surg Br.

[20] Boger A, Heini P, Windolf M (2007). Adjacent vertebral failure after vertebroplasty:a biomechanical study of low-modulus PMMA cement. Eur Spine J.

[21] Shinya N, Seiji T, Akihiro K (2009). Adjacent vertebral body fracture following vertebroplasty with polymethylmethacrylate or calcium phosphate cement. Spine.

[22] Pitton MB, Herber S, Bletz C (2008). Ct-guided vertebroplasty in osteoporotic vertebral fractures: incidence of secondary fractures and impact of intradiscal cement leakages during follow-up. Eur Radiol.

